# Curcumin attenuates harmful effects of arsenic on neural stem/progenitor cells

**Published:** 2017

**Authors:** Ali Jahanbazi Jahan-Abad, Parastoo Morteza-zadeh, Sajad Sahab Negah, Ali Gorji

**Affiliations:** 1 *Shefa Neuroscience Research Center, Khatam Alanbia Hospital, Tehran, Iran *; 2 *Department of Clinical Biochemistry, Shahid Beheshti University of Medical Sciences, Tehran, Iran*; 3 *Department of Biology, Kharazmi University of Tehran, Iran*; 4 *Department of Neuroscience, Mashhad University of Medical Sciences, Mashhad, Iran*; 5 *Epilepsy Research Center, Department of Neurology, and Department of Neurosurgery, Westfälische Wilhelms-Universität Münster, Münster, Germany*

**Keywords:** Medicinal plants, Toxicity, Stem cells, Neuronal injury

## Abstract

**Objective:**

Arsenic, an environmental pollutant, decreases neuronal migration as well as cellular maturation and inhibits the proliferation of neural progenitor cells. Curcumin has been described as an antioxidant and neuroprotective agent with strong therapeutic potential in some neurological disorders. Human adipose-derived stem cells (hADSCs), a source of multipotent stem cells, can self-renew and differentiate into neural cells. The aim of the present study was to investigate the preventive effect of curcumin against arsenic toxic effects on the viability, telomerase activity, and apoptosis of neural stem/progenitor cells (NSPCs) derived from hADSCs.

**Materials and Methods:**

The characteristics of human adipose tissue were identified by immunocytochemistry for surface markers namely, CD105, CD73, and CD90. Using neurosphere assay, hADSCs were differentiated into neuronal cells. To characterize neural cells, expression of nestin, SOX2, MAP2, and GFAP were assessed by immunocytochemistry. Cytotoxicity and viability of NSPCs were evaluated by MTT assay. Reactive oxygen species (ROS) generated by arsenic exposure, were measured and caspase 3/7 activity and caspase-3 processing as well as the telomerase activity were determined.

**Results:**

The isolated hADSCs positively expressed CD105, CD73, and CD90. Nestin, Sox2, GFAP, and MAP2 were expressed in the neurospheres derived from hADSCs. Curcumin/arsenic co-treatment significantly increased telomerase activity of NSPCs compared to arsenic group. Furthermore, curcumin significantly reduced arsenic-induced apoptosis (via inactivation of caspases) as well as arsenic-associated ROS generation.

**Conclusion:**

Our findings revealed that curcumin has the potential to prevent harmful effects of arsenic on neurogenesis.

## Introduction

Arsenic, an environmental pollutant, exists in soil and bedrock and may leach into ground- and surface-water (Andrade et al., 2015 ▶). Exposure to high levels of arsenic (i.e. via drinking water, corps, rice, etc.) puts a population of more than 100 million, mainly in developing countries, at risk of several serious illnesses (Shankar et al., 2014 ▶; Smith et al., 2000 ▶). Arsenic poisoning is a global concern especially in Southeastern Asia and Latin America. Clinically, arsenic affects several organs and may cause coronary and ischemic heart diseases, acute myocardial infarction, pulmonary tuberculosis, lung cancer, skin cancer, kidney cancer and endocrine system dysfunctions (Naujokas et al., 2013 ▶). It has been shown that arsenic could interfere with different cell processes, such as cell cycle, differentiation, and DNA repair and is able to modulate several signaling pathways. At molecular levels, the reactive oxygen species (ROS) generated due to arsenic exposure, interact with a large number of zinc-finger motif-containing proteins involved in DNA repair and therefore, lead to a break in single-strand and double-strand and cause mutations (Ho, 2004 ▶; Shi et al., 2004 ▶). Several investigations have shown that arsenic, even at non-toxic levels (Andersen., 2000 ▶), can lead to neuro-developmental effects in humans, including cognitive impairments, hearing loss, and peripheral neuropathy (Bencko et al., 1977 ▶; Dakeishi et al., 2006 ▶; Fincher et al., 1987 ▶). Similar neuro-developmental effects of arsenic have been reported in several animal studies, suggesting arsenic adverse effects on neural development during gestation (Hill et al., 2009 ▶; Tyler and Allan, 2013 ▶). Arsenic is strongly associated with aging, telomere shortening, and reduction in telomerase activity due to the generation of ROS. It has been shown that arsenic at low doses, inhibits neuronal formation from embryonic stem cells and leads to reduction in proliferation of neural progenitor cells and the number of mature neurons, possibly via the repression of neuron-specific transcription factors (Hong and Bain, 2012 ▶; Tyler and Allan, 2013 ▶). Therefore, suppressive effects of arsenic on embryonic stem cell differentiation may be an appropriate target for prevention of arsenic developmental impairments.

Usage of herbal compounds has received much attention in prevention of aging, cancer and neuronal disorders (Bakhtiari et al., 2016 ▶; Bakhtiari, 2015 ▶). Curcumin (diferuloylmethane), an active substance and yellow pigment obtained from the rhizoma of turmeric (*Curcuma longa*), has anti-inflammatory, antioxidant, anti-proliferative, anti-carcinogenic, and anti-tumor properties in different types of cell lines and animals and is widely used for metal detoxification (Chattopadhyay et al., 2004). It has been suggested that prevention of oxidative stress and induction of repair enzymes by curcumin, may be effective therapeutic strategies to reduce the adverse effects of arsenic (Roy et al., 2011 ▶). Mesenchymal stem cell is a potential tool for clinical treatment in the field of cell- and gene-therapy. One of the most crucial applications of adipose tissue-derived mesenchymal stem cells, is their differentiation into neural stem/progenitor cells (NSPCs) using neurosphere culture (Andersen, et al., 2000 ▶; Shankar and Shanker, 2014 ▶). Human adipose tissue-derived mesenchymal stem cells (hADSCs) are among mature stem cells. The purpose of the present investigation was to study the preventive effect of curcumin against arsenic effects on cell viability, telomerase activity, and apoptosis of NSPCs derived from hADSCs.

## Materials and Methods

In this study, adipose tissue was obtained from a 38-year old woman. Informed consent was obtained according to the ethical issues and the study was approved by the Ethics Committee of Shefa Neuroscience Research Center, Tehran, Iran.

**Isolation of mesenchymal stem cells from human adipose tissue**

For isolation of mesenchymal stem cells, the obtained adipose tissue was washed by phosphate buffered saline (PBS). Adipose tissue was finely minced with the help of a scalpel, digested using collagenase 0.075 % (Sigma- Aldrich) and incubated at 37^o^C for 45 min. Then, the digested adipose tissue was filtered using syringe filter. The dissociated tissue was centrifuged for 5 min at 400 g at 37^o^C. The supernatant was decanted and the pellet was re-suspended using Dulbecco's Modified Eagle Medium (DMEM, Invitrogen) supplemented with fetal bovine serum 20 % (FBS, Sigma- Aldrich) and seeded in T75 cm^2^ flasks. Adherent cells were observed after 24 hr.

**Immunophenotyping**

Immunocytochemistry was performed for surface markers CD105, CD73, CD90, CD56, and CD45 (Primary antibodies were purchased from Abcam) to identify the nature of the cells. For this purpose, 5×10^4^ cells were seeded in a 12-well plate. After 24 hr, when cells were attached to the plate, one step of washing with PBS was performed and paraformaldehyde 4% (Sigma- Aldrich) was used for 15 min at room temperature to fix the cells. The fixed cells were exposed to blocking buffer that contained FBS, for 5 min at 4^o^C to increase the specificity of the primary antibody. Cells were then incubated overnight at 4^o^C with anti-mouse primary antibodies. Fluoresce in Isothiocyante was used as a secondary antibody (Millipore). For 2 h at 4^o^C, cells were observed by inverted fluorescence microscope (Olympus IX71, Japan). For detection of nuclei, propidium iodide 100 µg/ml (Sigma- Aldrich) was used.

**Differentiation of hADSCs to neurosphere**

When cells reached 80-90% confluence, they were isolated through trypsinization and centrifugation. Cells were then cultured in T75 flasks using Dulbecco's modified essentialmedium with Ham's F-12 (DMEM/F12, Invitrogen) supplemented with basic fibroblast growth factor 20 ng/ml (bFGF, Sigma), B27 3% (Invitrogen), N2 1%, (Invitrogen) and epidermal growth factor 20 ng/ml (EGE, Sigma). After 4-6 days neurospheres were formed (Sahab Negah et al., 2016a ▶,b ▶).

**Production of neural precursor cells from neurospheres**

Neurospheres were centrifuged and the obtained pellet was re-suspended in DMEM/F12 medium supplemented with 5% FBS and bFGF, B27, N2, and EGE and cultured in T25 flasks coated with poly L-Ornithine and incubated for 2 weeks. To evaluate the neural cells, neural markers namely, nestin, GFAP, MAP2, and SOX2 were studied.

**Optimum time and concentration for curcumin and arsenic**

MTT assay was used to obtain the optimum concentrations and the optimal time for influence of both arsenic (Arsenic (III) oxide, Sigma-Aldrich, Germany) and curcumin (Sigma Chemical Co, St Louis MO, USA). According to the manufacturer instruction, curcumin was dissolved in DMSO to make a 15 mg/ml stock and stored at -20 °C. Then, an aliquot of the stock was further diluted in enriched DMEM to obtain the desired concentration. We first examined the cell viability at different concentrations of arsenic (III) oxide (0.1, 0.2, 0.5, 1, 2.5, and 5 mg/l) after 4, 12, 18, 24, 36 and 48 h exposure to arsenic. The same procedure was performed for the optimal concentration of curcumin at 1, 2, 5, 10, 20, and 50 µM.

**ROS generation**

A number of 3×10^5^ cells per ml was plated in 6-well plates and treated with curcumin 10 μM at the time of medium change. Then, Abcam's Cellular Reactive Oxygen Species Detection Assay Kit (Cambridge, UK) was used according to manufacturer’s instruction to quantitatively assess ROS production in control, arsenic-treated, curcumin-treated and arsenic-curcumin co-treated cells after 4, 8, and 12 hr.

**Caspase 3/7 assay**

To study the occurrence of apoptosis, caspase-Glo® 3/7 luminescent assay was used to measure caspase 3/7 activity. Cell lysate prepared by the cytosolic fractionation method with equal protein concentration was subjected to detect caspase-3/7 activity as described by manufacturer's instruction. Cells were harvested, washed with PBS, centrifuged and re-suspended in 400 ml of hypotonic buffer A (10 mM HEPES–KOH pH 7.5, 1.5 mM MgCl_2_ , 10 mMKCl, 1 mM Na-EDTA, 68 mM sucrose, and 1 mM PMSF). Subsequently, the cells were located on ice for 15 min, and then, vortexed for 30–45 sec. The cell debris was pelleted by centrifugation at 4°C, 14,600 g for 30 sec. For luciferase assay, the supernatant of cell lysate was used for luciferase assay. The activity was immediately recorded upon addition of 20 ml of luciferase assay complex (25 mM Tris, 10 mM MgSO_4_, 1 mM luciferin) (Resem BV, Netherlands) and 2 mM ATP (Roche, Germany) to 20 ml of cell lysate using a luminometer (Berthold Detection System, Germany)..

**Immunoblotting**

Immunoblotting was performed to further investigate the cleavage of caspase-3 in cytosol. Cells were lysed in Cell Culture Lysis Reagent buffer (Promega, Germany) containing 100 mM potassium phosphate (pH 7.8), 1 mM EDTA, 7 mM 2-mercaptoethanol, 1% (v/v) Triton X-100, and 10% (v/v) glycerol. Then, 100 μl of CCLR buffer was added to the collected cells and the mixture was stored at -80°C. To determine protein concentration in the cell lysate, standard Bradford method was used. After boiling for 5 min, 8% SDS polyacrylamide gels were prepared, and 50 µl samples were loaded into the wells. Gels were run for 90 min at 100 volt. Proteins were then transferred to a nitrocellulose membrane for 60 min by using a current of 250 mA. The membrane was blocked using blocking buffer for 1 hr at room temperature. Then, the membrane was incubated overnight at 4^o^C with anti-caspase 3 antibody (Abcam, UK), diluted at 1:1000. After incubation, the membrane was washed with PBS 0.05% tween solution three times and incubated for 1 hr at 1:1000 dilutions of horseradish peroxidase–conjugated goat anti-rabbit (Abcam, UK). Strips were washed three times with PBS 0.05% tween solution and the proteins of interest were detected using LI-COR imager.

**Detection of telomerase activity**

Telomerase activity was determined using the telomeric repeat amplification protocol (TRAP) with the TeloTAGGG PCR enzyme-linked immunosorbent assay ELISA kit (Roche, Germany) according to the manufacturer's instructions.

**Statistical analysis**

Comparisons between groups were made using Tukey's HSD *post-hoc* test with ANOVA. Results were considered statistically significant if p<0.05. Where appropriate, the data were also analyzed by one-way ANOVA.

## Results

**Isolation and characterization of hADSCs**

**Figure 1:**
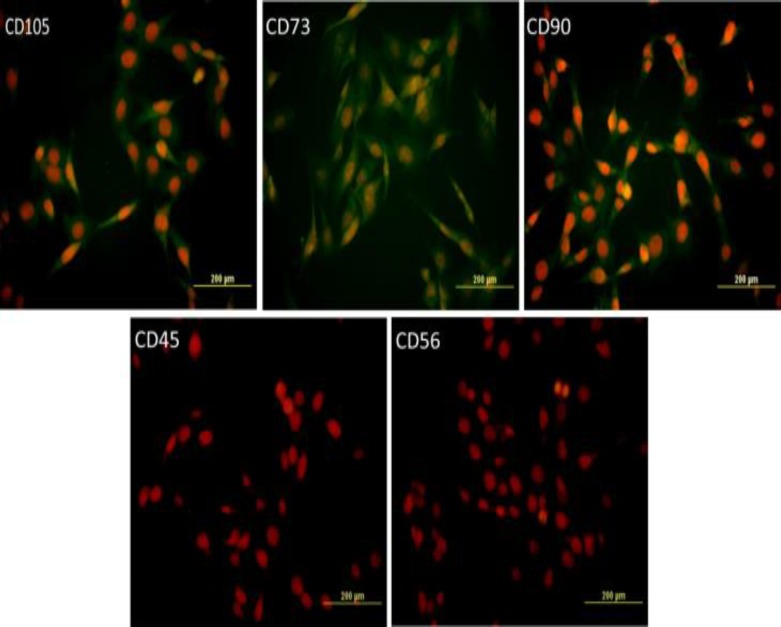
Immunocytochemistry staining of cell surface markers of human adipose tissue-derived from mesenchymal stem cells (hADSCs). Expression of markers CD105, CD73, CD90, CD45, and CD56 are presented (green). Nuclei were counterstained with DAPI. The expression levels of CD45 and CD56 were reduced compared to CD105, CD73, CD90. hADSCs was only expressed at low levels of CD45 and CD56

To confirm the mesenchymal nature of the cells, cell surface markers namely, CD73 ،CD90, CD105, CD45, and CD56 were investigated. hADSCs showed high expression of CD105, CD7 and CD90 (>98%) and low expression of CD45 and CD56 (<1.5%). These findings demonstrated the mesenchymal nature of the cells (Figure 1).

**Generation and differentiation of neurospheres from hADSCs**

**Figure 2:**
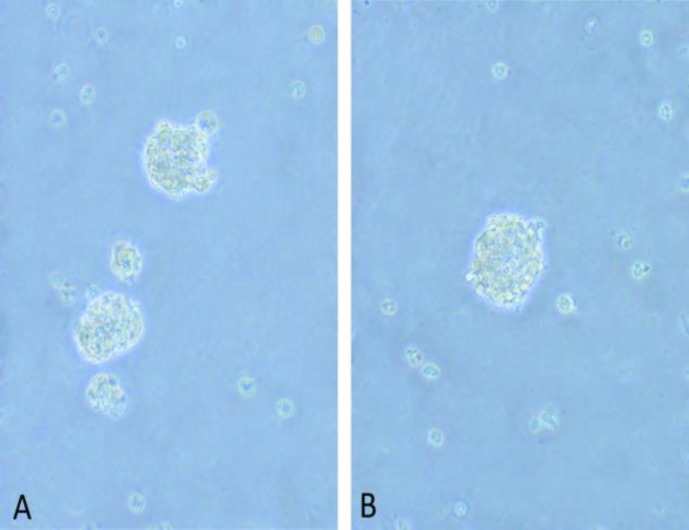
Neurosphere derived from ADSCs. A: Neurospheres were formed after 7 days. The size and shape of neurospheres were different. B: Secondary neurosphere was observed after the first passage

**Figure 3:**
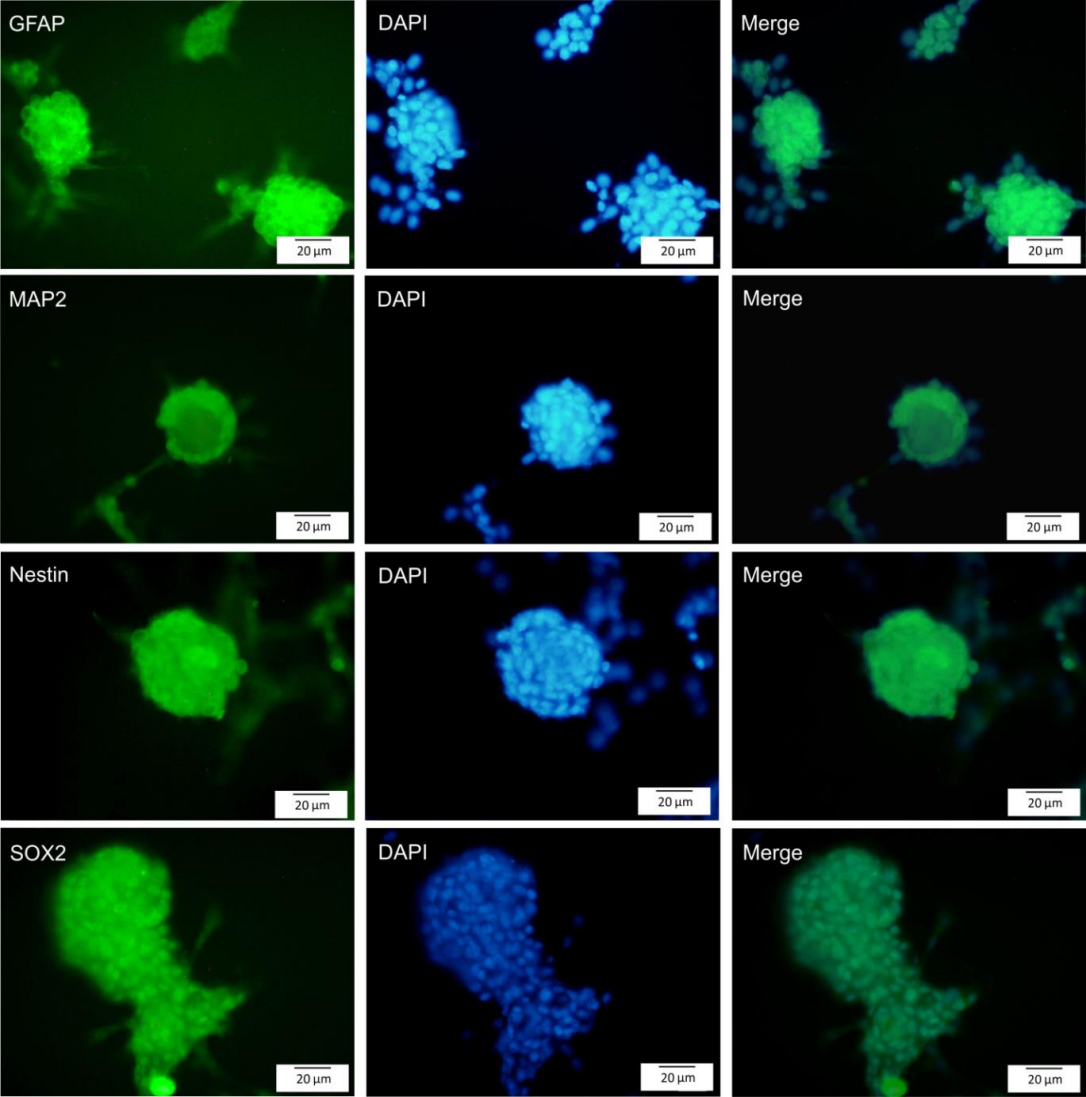
Immunocytochemistry staining of neuroshpere derived from hADSCs by using antibodies for GFAP, MAP2, nestin, and SOX2 (green). Nuclei were counterstained with DAPI. The merged images are also presented

**Figure 4:**
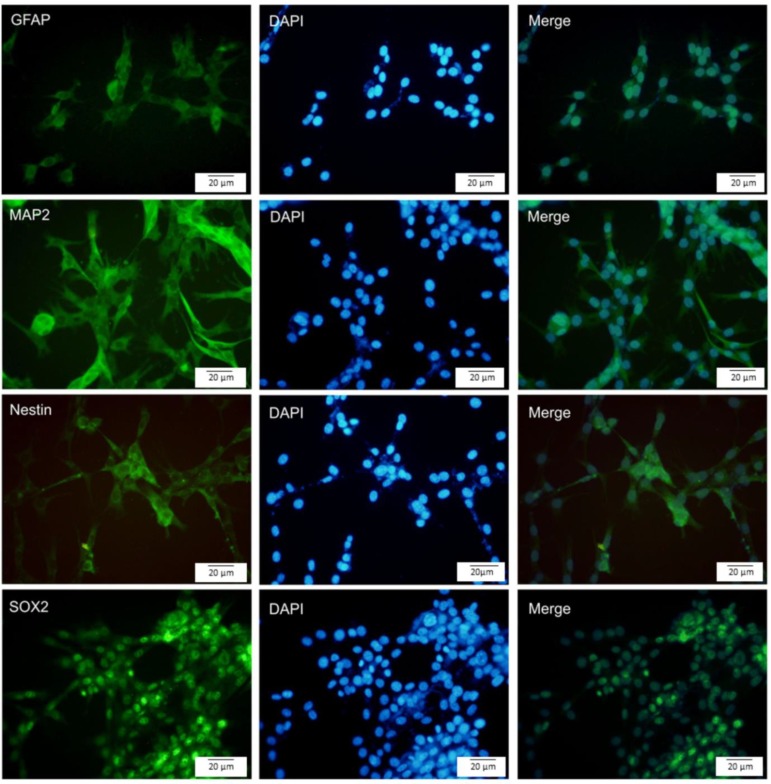
Immunocytochemistry staining of single cells derived from hADSCs by using markers for GFAP, MAP2, nestin, and SOX2 (green). Nuclei were stained with DAPI. The merged images are also shown. hADSCs expressed the mentioned markers

hADSCs were incubated in neurosphere medium for 2 weeks. Neurospheres were formed after 7 days (Figure 2). Primary neurospheres could proliferate and form secondary neurospheres after passaging. Characterization of neurospheres and single cells derived from hADSCs was done by immunocytochemistry*. *Neurospheres were stained with GFAP, MAP2, SOX2, and nestin. The neurospheres were positive for MAP2 (mature neuron), GFAP (astrocyte), and both SOX2 and nestin (neural stem/progenitor cells; Figure 3). We also assessed the expression of neuronal markers in single cells. Our result showed that GFAP, MAP2, SOX2, and nestin were expressed by single cells (Figure 4). 

**Dose- and time-dependent response of cell viability to arsenic and curcumin**

**Figure 5:**
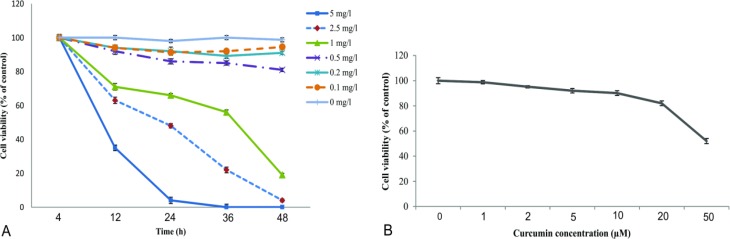
The optimal dose and duration of arsenic and curcumin application on neural stem/progenitor cells survival. A: Progenitor cells were treated with different concentrations of arsenic (0, 0.1, 0.2, 0.5, 1, 2.5 and 5 mg/l) and cell viability was measured at different intervals (4, 12, 24, 36 and 48 hr) after treatment. Here, arsenic 5 mg/l shows the highest mortality rate after 24 hr. B: Cells were treated with different concentrations of curcumin (0, 1, 2, 5, 10, 20 and 50 µM) and cell viability was measured after 24 h of incubation. Curcumin 10 µM was the highest concentration that showed the lowest effect on cell viability

To investigate the optimal dose and duration of arsenic application on cell viability, cell survival were measured after 4, 12, 24, 36, and 48 h of arsenic administration at concentrations of 0.1, 0.2, 0.5, 1, 2.5, or 5 mg/l. As illustrated in Figure 5A, exposure to arsenic at 5 mg/l significantly reduced the cell viability after 12 h of exposure to arsenic compared to the other doses and decreased the cell viability to 4% of the control level after 24 h. The same procedure was performed for testing the effect of different concentrations of curcumin (1, 2, 5, 10, 20, and 50 µM) on the cell viability after 24 h. As shown in Figure 5B, over 90% of cells were viable after treatment with curcumin at 10 µM. At higher concentrations of curcumin, the percentage of cell viability significantly decreased. Based on the abovementioned findings, the probable protective effect of curcumin 10 µM on adverse effects of arsenic 5 mg/l in NSPCs after 24 hours was tested.

**ROS generation in NSPCs after treatment with arsenic and/or curcumin**

**Figure 6:**
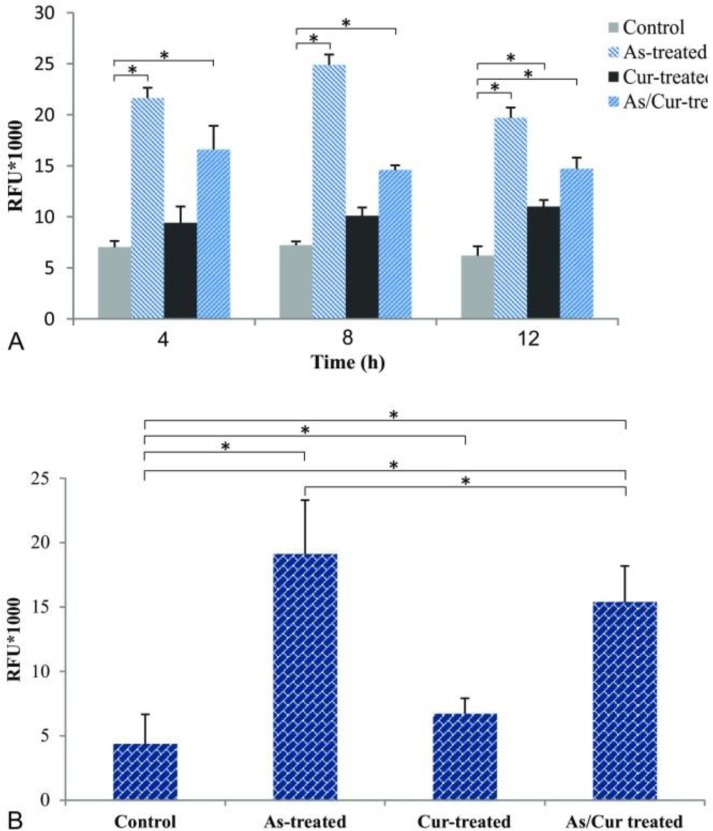
Reactive oxygen species (ROS) in neural stem/progenitor cells after treatment with arsenic and curcumin. ROS generation was measured at different intervals (4, 8 and 12 hr) after incubation of cells treated with arsenic, curcumin or both and compared to non-treated cells (control). * indicates significance at p<0.01

Administration of arsenic is strongly associated with the generation of ROS (Hosseinzadeh dehkordi et al., 2015 ▶). As shown in Figure 6A, an initial 3-fold increase in ROS generation occurred after 4 h of cell exposure to arsenic. The highest level of increased ROS was observed after 8 h of arsenic application (3.45 folds). In curcumin-treated NSPCs, ROS generation constantly increased by 1.2, 1.4, and 1.8 folds after 4, 8, and 12 h, respectively. Co-treatment with arsenic and curcumin, enhanced ROS production by 2.7, 2, and 2.3 folds after 4, 8, and 12 h, respectively, compared to the control group. There were significant differences between arsenic-treated and arsenic-curcumin treated groups in all measurements (p<0.05). ROS production significantly increased in curcumin-treated compared to control group after 12 h of exposure (p<0.05). Co-application of curcumin and arsenic significantly decreased ROS production by 23.1%, 41.3%, and 33.8% after 4, 8, and 12 hr, respectively, compared to administration of arsenic alone. As shown in Figure 6B, after 24 h of co-treatment with curcumin and arsenic, the level of ROS production was reduced to 19.5% compared to arsenic alone (p<0.05).

**Telomerase activity in NSPCs after treatment with arsenic and/or curcumin**

**Figure 7:**
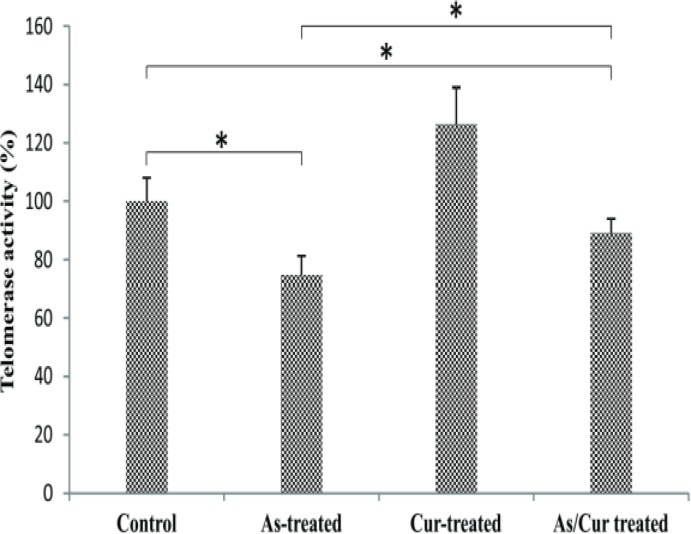
Telomerase activity of neural stem/progenitor cells after treatment with arsenic and curcumin. Telomerase activity was measured in control, arsenic-treated, curcumin-treated and arsenic/curcumin co-treated groups when cells were in passage 5. * indicates significant at p<0.01

Telomerase activity was measured after the 5^th^ passage. Application of curcumin increased telomerase activity by 26.3% compared to the control group (Figure 7). Arsenic caused 24.3% reduction in telomerase activity compared to control group (p<0.05). Co-administration of arsenic and curcumin, however, only decreased telomerase activity by 11% compared to the control group (p<0.05). Co-treatment with arsenic and curcumin significantly increased telomerase activity compared to arsenic alone (p<0.05; Figure 7). 

**Caspase 3/7 activity in NSPCs after treatment with arsenic and/or curcumin**

**Figure 8:**
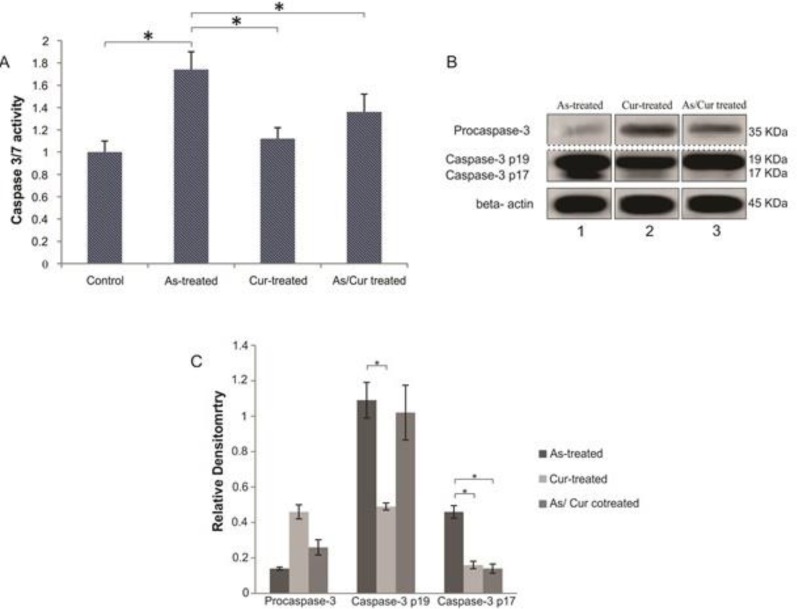
Caspase 3/7 activity of neural stem/progenitor cells after treatment with arsenic and curcumin. Caspase 3/7 activity was measured as an indicator of apoptosis in arsenic-treated, curcumin-treated and arsenic/curcumin co-treated groups compared to control group. The numbers show the fold changes relative to the control group.* indicates significance at p<0.01

To evaluate the rate of apoptosis in NSPCs, we measured the changes in caspase 3/7 activity. As illustrated in Figure 8A, treatment with arsenic significantly increased the activity of caspase 3/7 by 1.74 folds compared to the control group (p<0.05). Co-treatment with arsenic and curcumin significantly decreased caspase 3/7 activity compared to arsenic alone (p<0.05). Co-application of these two compounds raised caspase 3/7 activity only by 1.36 folds. Arsenic significantly increased the activity of caspase 3/7 compared to curcumin-treated group (p<0.05; Figure 8A).

**Figure 9.**
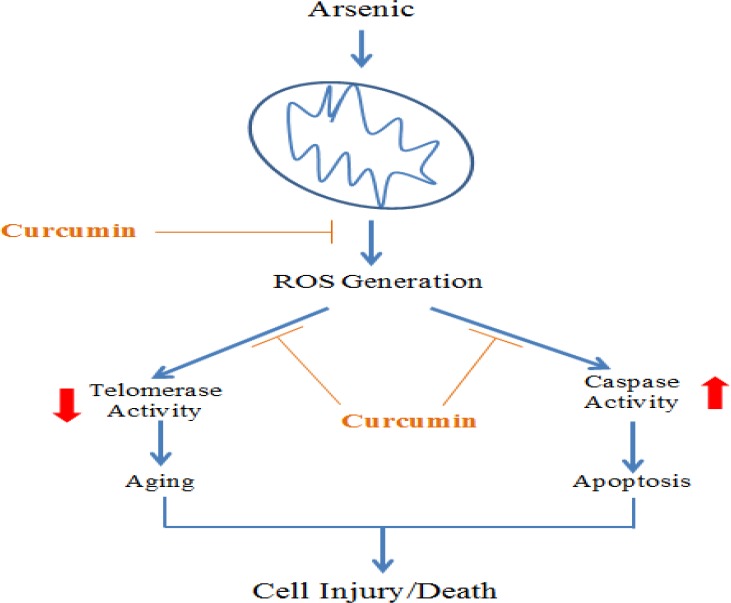
Schematic representation of the effect of Arsenic on cell damage/death and preventive role of curcumin. Exposure to arsenic leads to the generation of ROS through the interaction with mitochondria. ROS generation directs the cells toward apoptosis by enhancement of caspases activity. Furthermore, the production of ROS is associated with reduction of the telomerase activity. According to our results, curcumin inhibited the generation of ROS and the upstream events where it inhibited caspase activity and increased the telomerase activity

**Immunoblotting**

To further investigate the caspase-dependent apoptosis, immunoblotting assay of cleaved (active) caspases was performed for caspase-3. Application of arsenic led to cleavage of caspase-3 in NSPCs as indicated by prominent detection of the p17 and p19 fragments. Although caspase-3 cleavage occurred in curcumin and arsenic/curcumin-treated NSPCs, a part of caspases was still in the form of procaspase-3. Procaspase-3 was dominated in arsenic/curcumin treated NSPCs and caspase cleavage fragment was at lower density compared to that of arsenic-treated tissue (Figure 8B). Quantification of western blotting showed that caspase-3 cleavage (p17 and p19 fragments) significantly decreased in curcumin-treated group compared to arsenic group (p<0.01). Caspase-3 p17 was significantly lower in arsenic-curcumin co-treated group compared to arsenic group (p<0.001; Figure 8C).

## Discussion

Keeping in line with previous studies, our results indicated that neural stem/progenitor cells, astrocytes, and mature neurons could be obtained from the neurospheres derived from hADSCs (Kwon et al., 2011 ▶**; **Lim et al., 2010 ▶). Application of arsenic decreased the cell viability, enhanced ROS production and telomerase activity, and activated the caspase-dependent apoptosis. Simultaneous application of curcumin and arsenic inhibited arsenic-induced ROS production and prevented the activation of telomerase and caspase-dependent apoptosis.

In line with our findings, it has been shown that curcumin alleviates arsenic-induced cell damage in skin stem cell (Poojan et al., 2015 ▶), lung cancer cell lines (Hosseinzadeh dehkordi, et al., 2015 ▶), hepatic cells (Muthumani and Miltonprabu, 2015 ▶), kidney cells (Sankar et al., 2016 ▶) and the splenocytes (Khan et al., 2012 ▶), as well as protection against arsenic-induced genotoxicity (Sankar et al., 2014 ▶). Arsenic, as a pro-oxidant in different biological systems, can cause oxidative damage in the brain (Ramos et al., 1995 ▶). Naturally occurring antioxidants, such as curcumin, have been suggested to replace the conventional chelating agents for treatment of arsenic toxicity (Sankar, et al., 2016 ▶). Numerous investigations have demonstrated anti-oxidant and anti-inflammatory properties of curcumin (Menon and Sudheer, 2007 ▶), which may lead to neuroprotection and promotion of cell differentiation (Chen et al., 2014 ▶). Curcumin has been suggested to prevent arsenic-induced oxidative damage via reduction of oxidative stress and induction of repair enzymes (Roy, et al., 2011 ▶).

Arsenic induces ROS formation via activation of Nox2, a specific NADPH oxidase. This downregulates nitric oxidase synthase and leads to the accumulation of oxidants (Ellinsworth, 2015 ▶). In our study, the maximum ROS generation occurred after 8 h exposure to arsenic in NSPCs, which was significantly prevented by curcumin application. Curcumin also prevented arsenic-induced ROS formation in lung cancer cells (Hosseinzadeh dehkordi, et al., 2015 ▶). Arsenic causes DNA damage through enhancement of lipid peroxidation levels and ROS formation. Curcumin modulates ROS levels to maintain the physiological condition of the cells and prevent cell death (Fujisawa et al., 2004 ▶). Curcumin counteracts the cell damage via reduction of ROS production and lipid peroxidation and through enhancement of the phase II detoxification enzyme levels, such as catalase, superoxide dismutase, and glutathione peroxidase (Mukherjee et al., 2007 ▶).

High levels of arsenic have been reported to be associated with aging process in which telomere length and telomerase activity decrease (Zhan et al., 2003 ▶). Telomeres play a key role in the maintenance of chromosome integrity by protecting the ends of chromosomes from degradation and fusion (O'Sullivan and Karlseder, 2010 ▶). In contrast to somatic cells, telomerase activity is at very low level or even absent in most of stem cells regardless of their proliferative capacity (Hiyama and Hiyama, 2007 ▶). Arsenic exposure influences the telomere length, an effect that may be related to its carcinogenicity (Li et al., 2012 ▶). Our findings indicated that arsenic decreased telomerase activity, an effect which was counteracted by curcumin. 

Curcumin effect on increasing telomerase activity might be mediated via improving the stability of telomerase structure and in turn increasing the half-life of the enzyme (Xiao et al., 2014 ▶). Curcumin also enhanced DNA repair activity against arsenic-induced damages. The expression of polymerase, a repair enzyme, was found to be highly elevated when the damaged cells were permitted to repair in the presence of curcumin (Stankowska et al., 2015 ▶). Our findings revealed that cell damage and cell death in arsenic-treated NSPCs occurred through caspase-dependent apoptosis and curcumin diminished caspase processing. Curcumin reduced the vasoactive peptide endothelin-1-induced neuronal death of hippocampal neurons via inhibition of cleaved caspase-3 as well as reduction of caspases 3/7 activity (Stankowska, et al., 2015 ▶).

In conclusion, curcumin can be considered as a potential treatment to attenuate the harmful effect of ROS generation in arsenic-exposed neuronal stem cells. Furthermore, curcumin prevents the occurrence of apoptosis in neuronal stem cells via reduction of executioner caspase-3 activities and enhancement of telomerase activity (Fig. 9). This suggests that curcumin may be a promising tool to prevent neuro-developmental damages induced by arsenic exposure.
